# Supplementary material for: A common pathway for detergent-assisted oligomerization of Aβ42
Source: Commun Biol. 2023 Nov 21;6:1184. doi: 10.1038/s42003-023-05556-w (PMC10663524; doi:10.1038/s42003-023-05556-w)
Supplement: Supplementary file 2 — Supplementary Information [file 42003_2023_5556_MOESM2_ESM.pdf]

Stabilization of an Aβ42 Tetramer in Detergent Micelles

Fidha Nazreen Kunnath Muhammedkutty[1], Ramesh Prasad[1], Yuan Gao[2], Tarunya Rao Sudarshan[2], Alicia S. Robang[2], Jens O. Watzlawik[3], Terrone L. Rosenberry[3], Anant K. Paravastu[2], and Huan-Xiang Zhou[1,4]

[1]Department of Chemistry, University of Illinois Chicago, Chicago, IL, USA,

[2]School of Chemical and Biomolecular Engineering, Georgia Institute of Technology, 311 Ferst. Drive NW, Atlanta, GA 30332, USA

[3]Department of Neuroscience and Pharmacology, Mayo Clinic, Jacksonville, FL 32224, USA

[4]Department of Physics, University of Illinois Chicago, Chicago, IL, USA

*Correspondence should be addressed to H.X.Z. (e-mail: hzhou43@uic.edu) or A.K.P. (e-mail: anant.paravastu@chbe.gatech.edu).

Supplementary Information

Supplementary Table 1. Information for the systems studied by molecular dynamics simulations

Peptide	Parallel (p) or anti-parallel (ap)	Dimer (d) or tetramer (t)	SDS or DPC	N- or C-sheet	# of detergents	# of water molecules	# of atoms	Simulation box (Å^3)	Simulation time /replicate[a] (μs)
Aβ42	p	d	SDS	N	80	34857	109311	100×100×120	1
Aβ42	p	t	SDS	N	74	34186	108298	100×100×120	1
Aβ42	p	d	SDS	C	59	35308	109721	100×100×120	1
Aβ42	ap	d	SDS	C	63	39930	123803	80×120×140	1.2
Aβ40	p	d	SDS	C	62	35276	109736	100×100×120	1
Aβ42	p	t	SDS	C	62	34402	108430	100×100×120	2
Aβ40	p	t	SDS	C	61	33610	107959	100×100×120	1.1
Aβ42	ap	t	SDS	C	70	39043	122703	80×120×140	2.2
Aβ42	ap	t	SDS	C	61	55995	174288	100×130×160	1
Aβ42	ap	t	DPC	C	71	56269	175722	100×130×160	1.5 (2.0[b])
Aβ42	ap	t	DPC	C	61	57188	176771	100×130×160	1 (1.8[b])

[a]Four replicate simulations were run for each system.

[b]One of the replicate simulations was extended to the longer time.

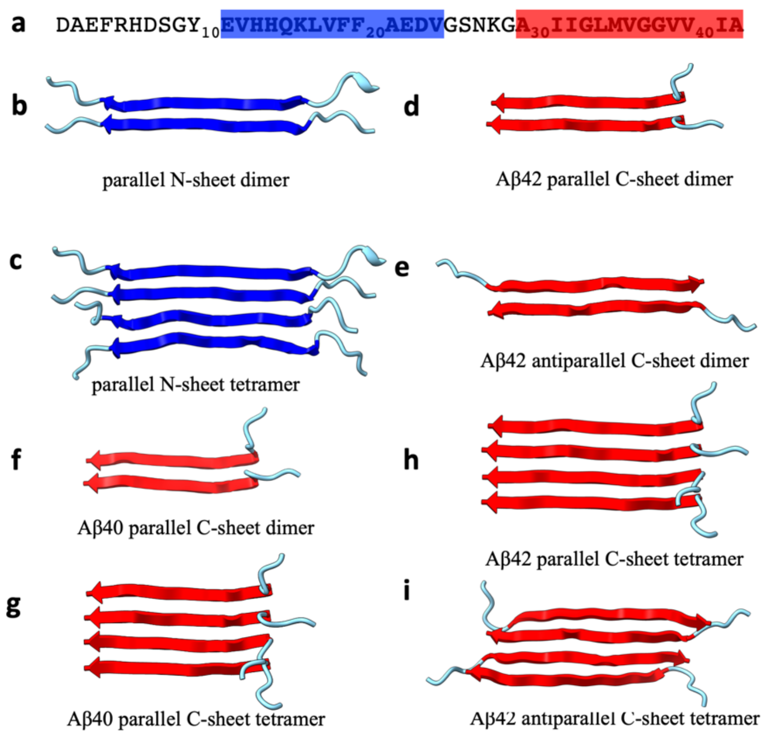

Supplementary Figure 1. Aβ sequence and oligomers studied here. (a) Aβ42 sequence, with N and C-strand highlighted in blue and red respectively. (b-i) Dimeric or tetrameric parallel or antiparallel N or C-sheet.

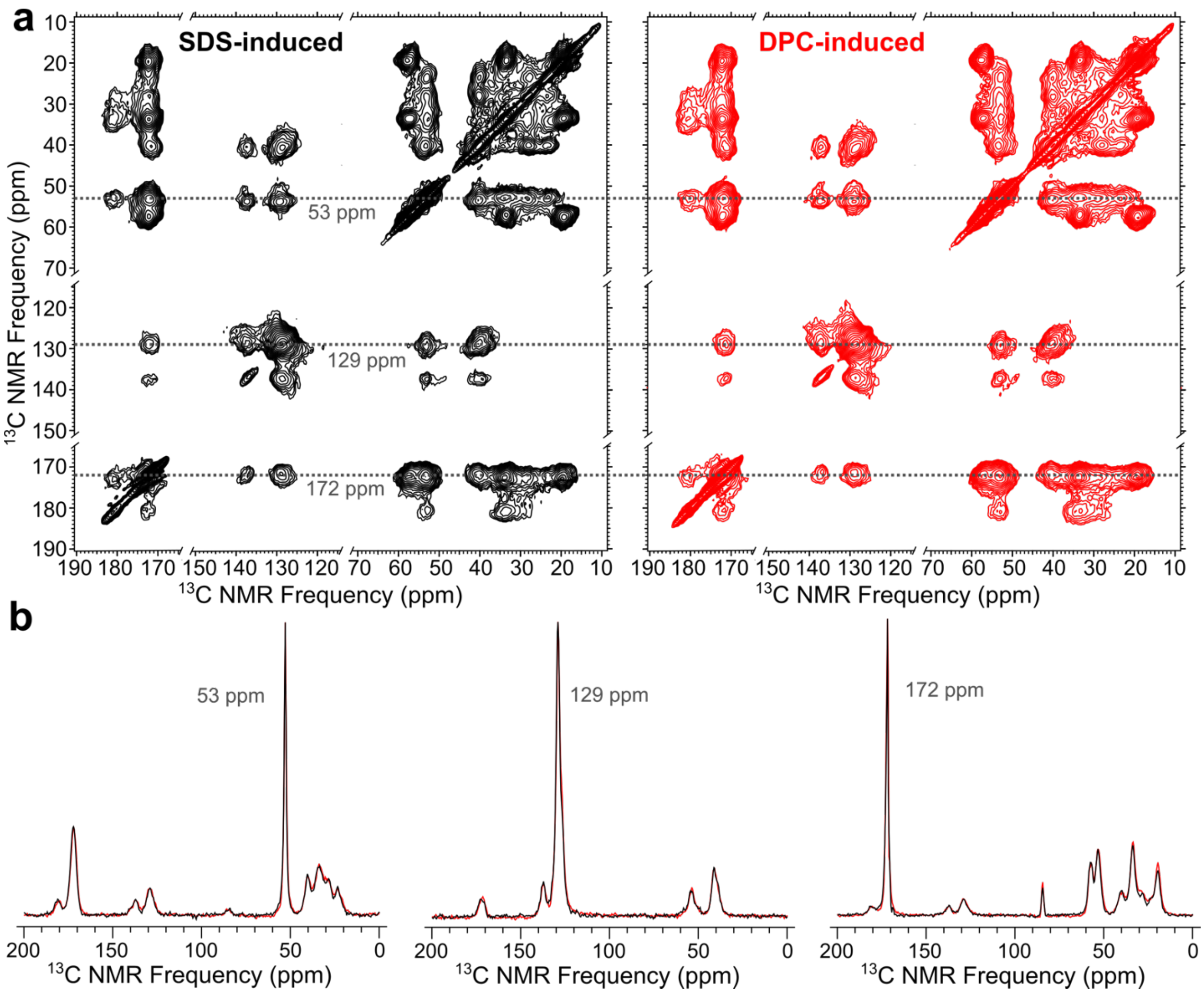

Supplementary Figure 2. Different presentations of the ^{13}C-^{13}C correlation spectra shown in Figure 2a. (a) Side-by-side comparison of the two spectra. (b) Three 1D slices, each as an overlay between the two spectra in close superposition (black and red curves for the SDS- and DPC-induced oligomers, respectively).

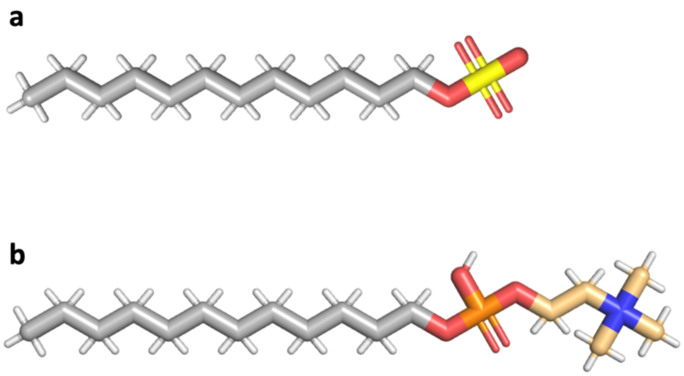

Supplementary Figure 3. SDS and DPC molecules have the same hydrocarbon tail but different head groups. (a) SDS molecule. (b) DPC molecule. The colors of headgroup atoms are: S, yellow; P, orange, O, red, N, blue, C, light orange.

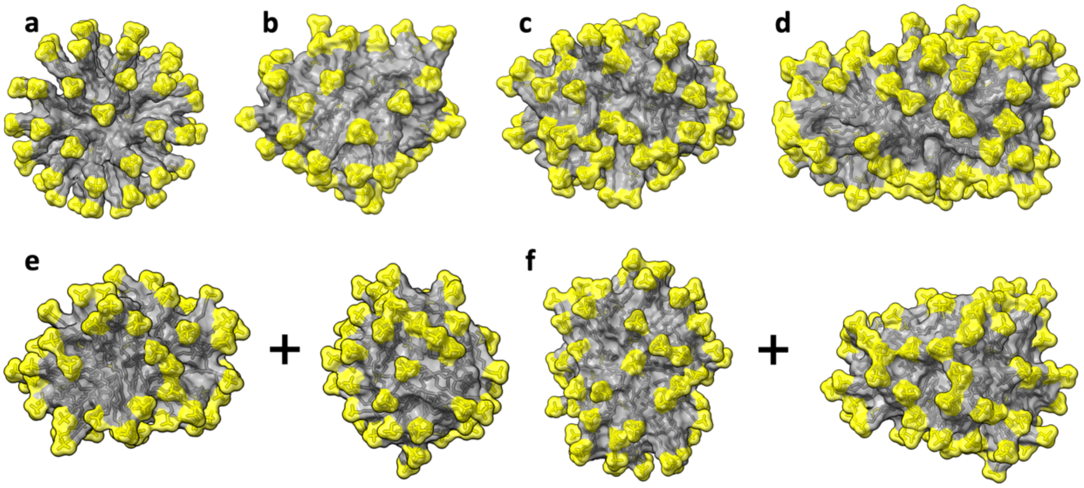

Supplementary Figure 4. MD simulations of pure SDS micelles. (a) Initial structure of a micelle with 60 SDS molecules. (b-f) Final structures of micelles with 60, 80, 100, 120, and 150 SDS molecules. In the last two cases, the initial micelle broke into two separate micelles. Each simulation was run for 500 ns. Micelles are rendered in surface representation, with headgroups in yellow and hydrocarbon tails in grey.

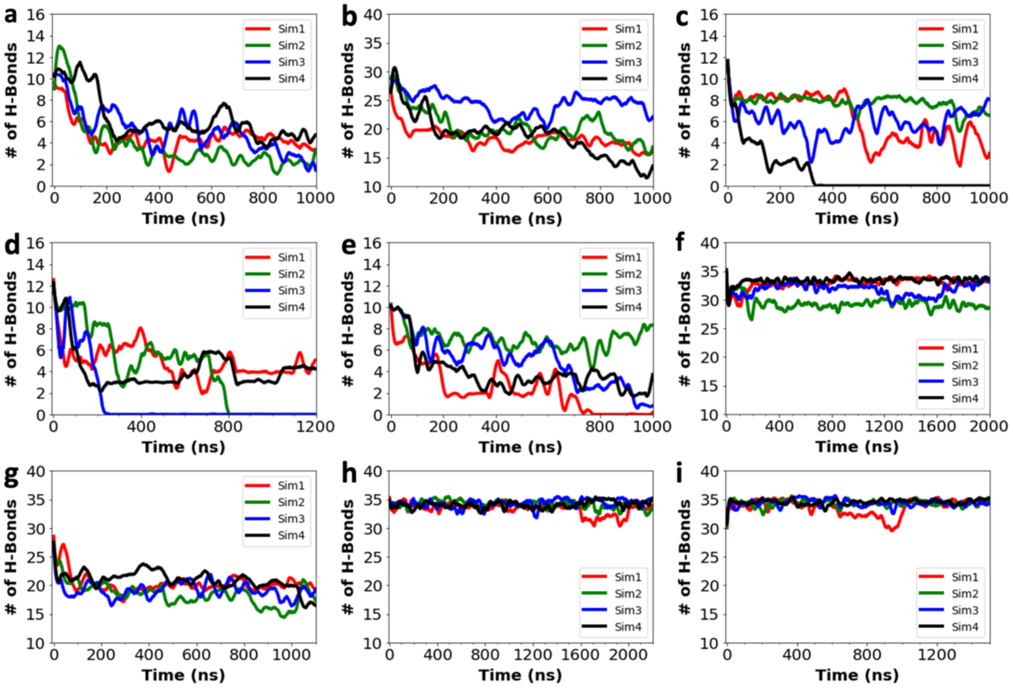

Supplementary Figure 5. Time traces of the numbers of backbone hydrogen bonds. (a) Aβ parallel N-sheet dimer. (b) Aβ parallel N-sheet tetramer. (c) Aβ42 parallel C-sheet dimer. (d) Aβ42 antiparallel C-sheet dimer. (e) Aβ40 parallel C-sheet dimer. (f) Aβ42 parallel C-sheet tetramer (g) Aβ40 parallel C-sheet tetramer. (h) Aβ42 antiparallel C-sheet tetramer. All the above are in SDS micelles. (i) Aβ42 antiparallel C-sheet tetramer in a DPC micelle.

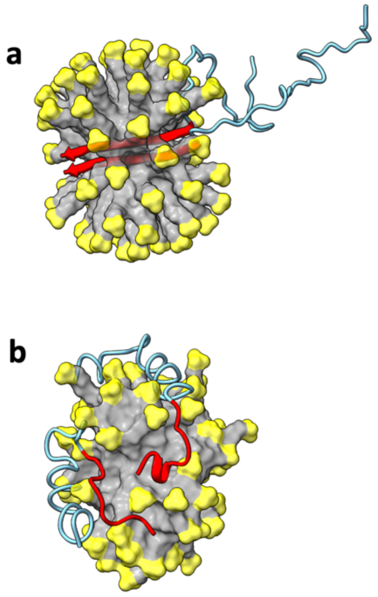

Supplementary Figure 6. MD simulations of the Aβ40 parallel C-sheet dimer in an SDS micelle. (a) Initial structure. (b) Snapshot at 1000 ns of a simulation. The SDS micelle is shown in surface representation with headgroups in yellow and hydrocarbon tails in grey. Aβ40 molecules are shown in cartoon representation with residues 1-29 in cyan and residues 30-40 in red.

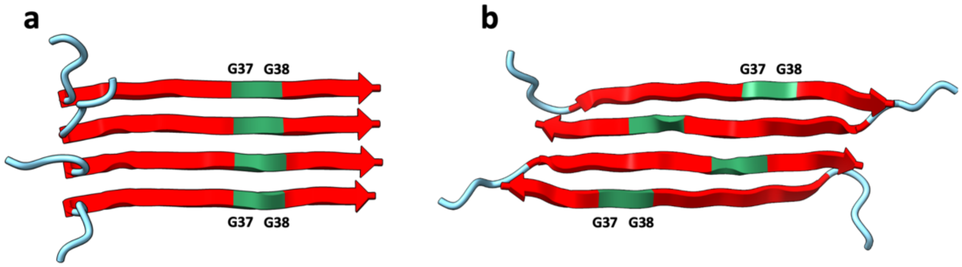

Supplementary Figure 7. Placements of G37 and G38 in parallel and antiparallel C-sheet tetramers of Aβ42. (a) Parallel C-sheet tetramer. (b) Antiparallel C-sheet tetramer. Aβ42 molecules are shown in cartoon representation with G37 and G38 in green, other residues in the C-strands in red, and the N-terminal residues in cyan.

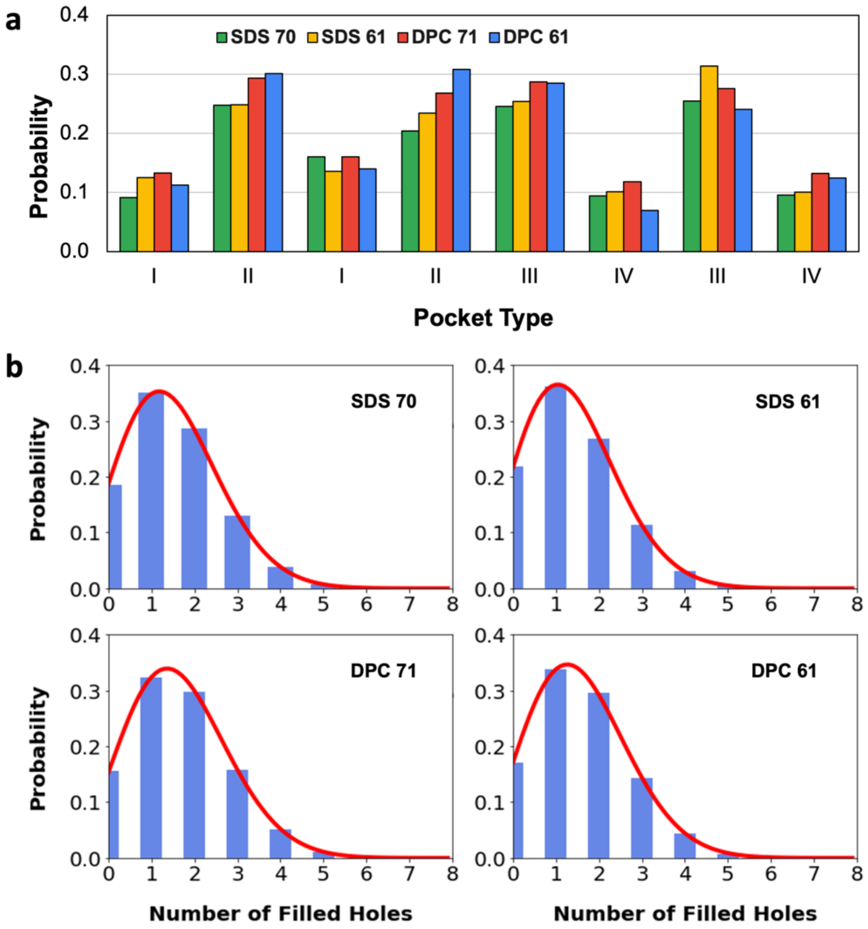

Supplementary Figure 8. Probabilities of glycine holes being filled by SDS or DPC molecules. (a) Probabilities of individual holes being filled in simulations with SDS and DPC micelles. The legend indicates the detergent molecule and micelle size; e.g., "SDS 70" means a micelle with 70 SDS molecules. (b) Probabilities for a certain number of glycine holes being simultaneously filled at a given time, in four different micelles indicated by the legends. Bars show the raw data; red curves show a binomial distribution, with the probability of a hole being filled given by the average among the eight holes.

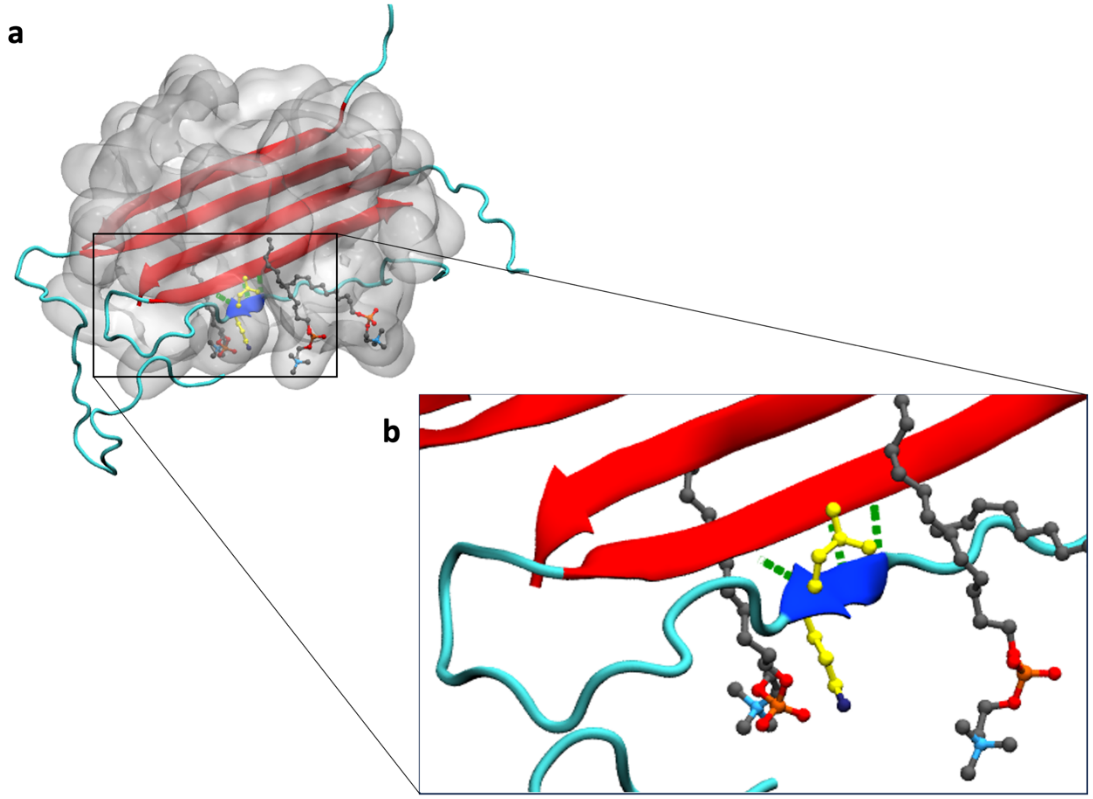

Supplementary Figure 9. The N-terminal region of an edge chain forms an additional β-strand docked to the antiparallel C-sheet of the Aβ42 tetramer in a micelle with 61 DPC molecules. (a) Snapshot at 162 ns of simulation 3. (b) Zoomed view showing the initiation of the N-strand docking to the edge C-strand. The C-sheet is shown in red; the N-strand is shown in blue; the DPC micelle is shown as a grey surface. Shown in ball-and-stick are the two residues, Lys16 and Leu17, that aid in the N-strand docking (carbon in yellow; nitrogen in dark blue) and the DPC molecules that interact with them (carbon in grey, oxygen in red, phosphorous in orange, nitrogen in light blue). Hydrogen bonds between the edge C-strand and the N-strand are shown as green dashed lines.

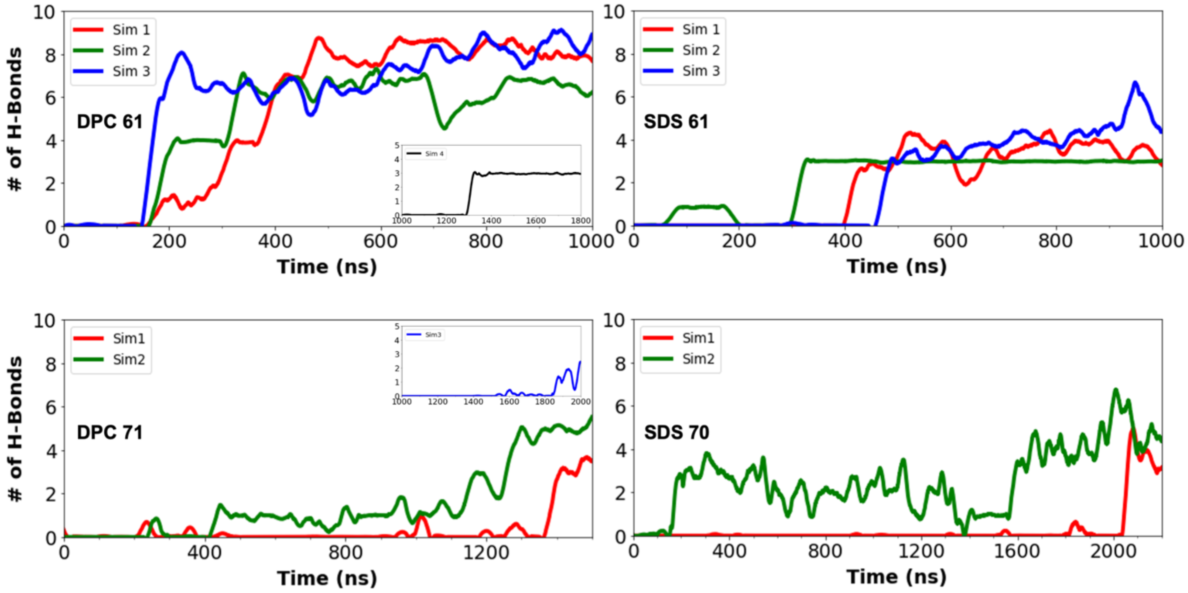

Supplementary Figure 10. Number of hydrogen bonds between the N-terminal region and the C-sheet, formed in micelles with 61 and 71 DPC molecules or with 61 and 70 SDS molecules.

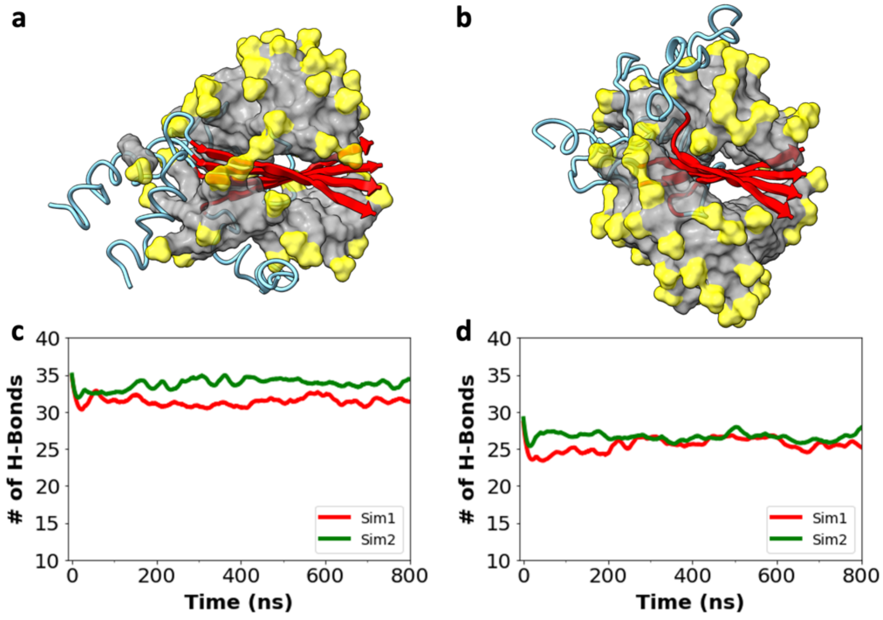

Supplementary Figure 11. Parallel C-sheet tetramers of Aβ42 and Aβ40 with the G37V and G38V mutations are stable in SDS micelles. (a) Aβ42 mutant tetramer at 702.0 ns of a simulation. (b) Aβ40 mutant tetramer at 710.1 ns of a simulation. (c) Time traces of the number of backbone hydrogen bonds in two replicate simulations of the Aβ42 mutant tetramer. (d) Corresponding results for the Aβ40 mutant tetramer.

Supplementary Movie 1. A 900-ns clip from simulation 1 of the Aβ42 antiparallel C-sheet tetramer in a micelle with 61 DPC molecules. A total of 901 frames were played at 10 frames per second; the 0th frame was the initial structure; the next four frames were from the equilibration stage; and the remaining frames were from the production stage. Frames were separated by 1 ns in simulation time. The C-sheet is shown in red; the N-strand that docks to the C-sheet is shown in blue; an α-helix formed in the N-terminal region of another chain is shown in green; the DPC micelle is shown as a grey surface. Shown in ball-and-stick are the two residues, Lys16 and Leu17, that aid in the N-strand docking (carbon in yellow; nitrogen in dark blue) and the DPC molecules that interact with them (carbon in grey, oxygen in red, phosphorous in orange, nitrogen in light blue). Hydrogen bonds between the edge C-strand and the N-strand are shown as green dashed lines.

Supplementary Movie 2. A zoomed clip from Supplementary Movie 1, from 100 ns to 200 ns, highlighting the initiation of N-strand docking to the edge C-strand.
