# Supplementary material for: A common pathway for detergent-assisted oligomerization of Aβ42
Source: Commun Biol. 2023 Nov 21;6:1184. doi: 10.1038/s42003-023-05556-w (PMC10663524; doi:10.1038/s42003-023-05556-w)
Supplement: Supplementary file 3 — Description of Supplementary Materials [file 42003_2023_5556_MOESM3_ESM.docx]

**Description of Additional Supplementary Files**

**File name:** Supplementary Movie 1

**Description:** A 900-ns clip from simulation 1 of the Aβ42 antiparallel C-sheet tetramer in a micelle with 61 DPC molecules. A total of 901 frames were played at 10 frames per second; the 0th frame was the initial structure; the next four frames were from the equilibration stage; and the remaining frames were from the production stage. Frames were separated by 1 ns in simulation time. The C-sheet is shown in red; the N-strand that docks to the C-sheet is shown in blue; an a-helix formed in the N-terminal region of another chain is shown in green; the DPC micelle is shown as a grey surface. Shown in ball-and-stick are the two residues, Lys16 and Leu17, that aid in the N-strand docking (carbon in yellow; nitrogen in dark blue) and the DPC molecules that interact with them (carbon in grey, oxygen in red, phosphorous in orange, nitrogen in light blue). Hydrogen bonds between the edge C-strand and the N-strand are shown as green dashed lines.

**File name:** Supplementary Movie 2

**Description:** A zoomed clip from Supplementary Movie 1, from 100 ns to 200 ns, highlighting the initiation of N-strand docking to the edge C-strand.
